# Quantum-dot assisted spectroscopy of degeneracy-lifted Landau levels in graphene

**DOI:** 10.1038/s41467-020-17225-1

**Published:** 2020-07-08

**Authors:** Itai Keren, Tom Dvir, Ayelet Zalic, Amir Iluz, David LeBoeuf, Kenji Watanabe, Takashi Taniguchi, Hadar Steinberg

**Affiliations:** 1 Racah Institute of Physics, The Hebrew University, 91904 Jerusalem, Israel; 2 LNCMI, Centre National de la Recherche Scientifique, EMFL, Université Grenoble Alpes, INSA Toulouse, Université Toulouse Paul Sabatier, Grenoble, France; 3 National Institute for Material Science, 1-1 Namiki, Tsukaba, 305-0044 Japan

**Keywords:** Electronic properties and materials, Quantum Hall

## Abstract

Energy spectroscopy of strongly interacting phases requires probes which minimize screening while retaining spectral resolution and local sensitivity. Here, we demonstrate that such probes can be realized using atomic sized quantum dots bound to defects in hexagonal Boron Nitride tunnel barriers, placed at nanometric distance from graphene. With dot energies capacitively tuned by a planar graphite electrode, dot-assisted tunneling becomes highly sensitive to the graphene excitation spectrum. The spectra track the onset of degeneracy lifting with magnetic field at the ground state, and at unoccupied excited states, revealing symmetry-broken gaps which develop steeply with magnetic field - corresponding to Landé *g* factors as high as 160. Measured up to *B* = 33 T, spectra exhibit a primary energy split between spin-polarized excited states, and a secondary spin-dependent valley-split. Our results show that defect dots probe the spectra while minimizing local screening, and are thus exceptionally sensitive to interacting states.

## Abstract (web summary)

Here, the authors develop a spectroscopic technique whereby individual defects in an ultrathin hBN dielectric, placed in proximity to graphene, act as quantum dots. Dot-assisted tunneling is highly sensitive to the nearby graphene excitation spectrum, and allows probing of energy splitting in the excited Landau levels.

## Introduction

The use of electron tunneling as a spectroscopic probe for condensed matter systems was first demonstrated by Giaever^1^, who applied an oxide tunnel barrier to map the gap in the excitation spectrum of superconductors. Tunneling measurements involve a source electrode which couples to a sample system through a barrier—with the sample density of states (DOS) encoded in the differential change in the tunnel current at a finite source bias^2^. Tunneling became a generic probe, able to address a broad range of conducting samples, following the introduction of the scanning tunneling microscope (STM)^3^.

Certain types of samples, however, challenge the existing tunneling methodology. For samples with low DOS, applied bias voltage leads to local charging effects, enhanced by differences in the work functions of the probe and sample. In graphene, for example, voltage applied to a local tunnel-probe changes the potential landscape^4–7^. At finite magnetic fields where graphene DOS becomes sharply peaked at Landau level energies ${E}_{N}=\pm\! {v}_{{\rm{F}}}\sqrt{2\hslash e| N| B}$^8,9^ (*N* being level index), deformation of the potential isolates local regions due to incompressible strips^5^. This is further complicated by the need to reach elevated Landau levels, requiring bias voltages reaching well over 100 mV.

An ideal probe would be local in its physical extent, minimize screening of local interactions and at the same time retain a parallel geometry to avoid non-homogeneous charging. In addition, it should sustain a high bias without deforming local potentials. Here we show that these seemingly contradicting requirements are fulfilled by resonant tunneling through quantum dots (QDs) bound to atomic defects within van der Waals tunnel barriers^10,11^. Graphene-based tunnel junctions are highly parallel^12^, and dot energies are capacitively tuned by the planar electrode, thus avoiding local charging effects due to applied bias. Barrier-embedded dots are nanometric—both in their physical dimensions, and in their proximity to the sample layer. Thus, they are sensitive to regions few nm large. Finally, defect dots lack any degree of freedom for charge rearrangement, they do not screen local interactions, and are hence less invasive than metallic probes.

When QDs couple weakly to the source and drain electrodes, they permit resonant charge transport through sharply peaked energy levels. In this regime, sample DOS is probed by the current through the dot, rather than differential current—avoiding large DC contributions. QDs are utilized as local thermometers^13,14^, where energy distribution is tracked using the energy-selective nature of injection and ejection of carriers through the QD. Alternatively, by electrostatic tuning of the QD level, sequential tunneling through the QD singles out the sample DOS in resonance with the dot—as seen in the gate-tunable dots used to probe the spectra of superconductor-proximitized nanowires^15,16^.

We demonstrate the utility of QD-assisted spectroscopy in a study of the graphene excitation spectrum. In the graphene quantum Hall regime, Landau levels are fourfold degenerate. This SU(4) symmetry allows for several distinct paths of symmetry breaking^17–22^ driven by magnetic field, causing the emergence of ordered ground states which may break either spin or valley degeneracy. The order in which these symmetries should break is subject to debate: While the spin degeneracy is broken by the Zeeman effect, the breaking of valley degeneracy via magnetic field is not as straight-forward. It appears to depend on sample-specific properties such as disorder and the effect of interactions^23^. Specifically, *N* = 0 and *N* ≠ 0 Landau levels differ in wavefunction localization, causing a difference in the energy splitting due to lifting of the valley degeneracy. The magnitudes of the Zeeman effect and the short range interactions compete, resulting in different hierarchies between the spin and valley degeneracy lifting.

So far, existing experiments sensitive to degeneracy lifting effects were provided by probes sensitive to the ground state. These include transport^23–25^ and STM measured near the Fermi energy^26,27^. The nature of degeneracy lifting in excited states remains an open question: A two-fold splitting of the filled *N* = 0 level has been observed by STM^26^, and it is indeed clear that excited energy levels should retain the Zeeman splitting. In excited state spectroscopy, carriers are injected into non-populated levels, or ejected from fully populated levels. In this scenario, any deviation from single-particle Zeeman splitting would indicate that energy levels are affected by inter-Landau-level interactions. A non-trivial role of interactions will be manifest in two ways. First, any enhancement of the Landé *g* factor from the non-interacting value, and second, the appearance of valley splitting in full or empty Landau levels.

## Results

### Defect-assisted tunneling at zero magnetic field

In this work we report measurements of defect-assisted transport between graphene and graphite separated by a hBN barrier. The barrier-defect energy is tunable by an electric field which originates from a top-gate and penetrates through the graphene layer. We carry out measurements up to magnetic fields of *B* = 33 T, and find an intricate pattern of lifting of both valley and spin degeneracies upon injection of carriers to the *N* = 0 and *N* = 1 excited Landau levels. The spectral splitting is dominated by a strongly enhanced Zeeman term, and valley-split energies are found to exhibit spin-valley coupling.

**Fig. 1:**
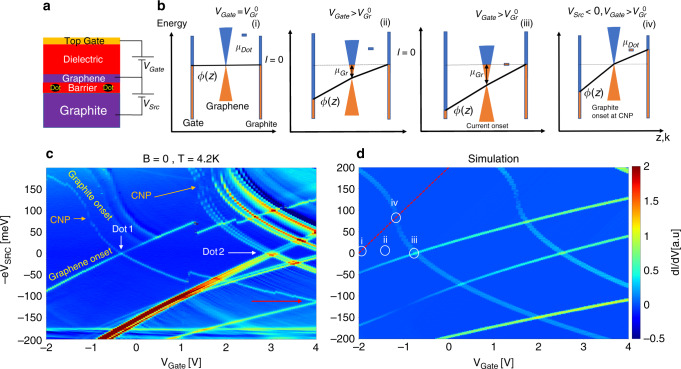
**Tunneling through quantum dots**. **a** A schematic illustration of the device. The quantum dots are present within the hBN layer between graphite and graphene. **b** The energy diagram illustrating the change of the electrostatic potential *ϕ*(*z*) upon application of *V*_Gate_ and *V*_Src_ and its effect on the dot potential *μ*_Dot_. The horizontal axis marks both *k* (momentum of the graphene dispersion) and *z* (the position coordinate). **c** d*I*/d*V* vs.  −e*V*_S*rc*_ and *V*_Gate_ at *T* = 4.2 K. Transport signatures of distinct quantum dots, “Dot 1” and “Dot 2” are marked. CNP is marked by an arrow for both dots. Graphite (source) onset and graphene (drain) onset lines are labeled for Dot 1 while the red arrow signals the charging energy of Dot 1. **d** Simulated d*I*/d*V* for the capacitive model described in the text, assuming two dots where *μ*_0_ = 40 meV for Dot 1 and *μ*_0_ = 67 meV for Dot 2. The positions corresponding to diagrams (i)–(iv) in **b** are marked and so is an equal density line, corresponding to *n*_G*r*_ = 0 near Dot 1 (red dashed line).

Defects are regularly found in exfoliated materials, and their signatures have been observed via photoluminescence in transition metal dichalcogenides (TMD)^28,29^ and hexagonal Boron Nitride (hBN)^30^ layers. Coupling defect-dots to source and drain electrodes entails placing an insulating layer between two conductors-the same geometry used for tunnel junctions stacked using the vdW transfer technique^31,32^ (Fig. 1a). This results in single charging behavior characteristic of quantum dots, as seen both in hBN and TMDs^10,33–35^. The dimension of a dot embedded within barriers depends on the type of defect and dielectric properties of the medium, and can range from the atomic size to a few nm^34,36^. In addition, being embedded in a few layer insulator, barrier defects reside at nanometer proximity to both source and drain.

We report measurements taken on a device fabricated using the standard polycarbonate (PC) pickup method, schematically depicted in Fig. 1a (See more details in Supplementary Note 5). The bottom (source) electrode is a graphite flake onto which a tunnel barrier hBN flake of thickness *d*_S*rc*_ = 2 nm (five layers) is deposited. Graphene is picked up and placed on top of the barrier, capped by a second 20 nm hBN flake. Ti–Au electrodes are deposited on the graphene and graphite, respectively, and a top-gate is patterned over the top hBN. Upon application of bias voltage *V*_Src_ between the graphite and graphene flakes, the differential conductance d*I*/d*V* measured at *T* = 4.2 K is dominated by several sharp features, which also depend on the gate voltage *V*_Gate_. *V*_Src_ and *V*_Gate_ both charge the graphene layer, adding a global charge density Δ*n*, such that *n*(*x*) =  *n*_0_(*x*) + Δ*n*(*x*) where *n*(*x*) is the local density and *n*_0_(*x*) is the local density at *V*_Gate_  = *V*_S*rc*_ = 0. Δ*n* is calculated using the capacitive coupling of graphene to the source and gate.

A plot of d*I*/d*V* vs.  −e*V*_Src_ and *V*_Gate_ is presented in Fig. 1c. It exhibits sharp features reminiscent of Coulomb-blockade diamonds, interpreted as the onset of resonant tunneling conductance through quantum dots embedded within the hBN barrier. It is immediately evident that the slopes of the differential conductance features in Fig. 1c are not constant. Such slopes are determined by the ratios of respective dot capacitances to the source, drain and gate electrodes^10^. The gate, however, is separated from the dot by the graphene layer, which screens its electric field. As this screening varies with the graphene density, the effective dot-gate capacitance varies as well. Field penetration through graphene is explained in the energy diagrams in Fig. 1b, where we schematically plot the evolution of graphene chemical potential *μ*_Gr_, electrostatic potential *ϕ*(*z*), and dot chemical potential *μ*_Dot_ with respect to applied *V*_Gate_ and *V*_Src_. (Here *z* is the spatial coordinate perpendicular to graphene surface, with *z* = 0 the position of the graphene layer. *ϕ* and *μ*_Gr_ are defined as positive for electrons).

### Capacitive model for defect-assisted tunneling

We take as a starting condition the neutrality point where graphene density is *n*_Gr_ = 0, *V*_Src_ = 0. At this condition we define ${V}_{{\rm{G}}{\mathrm{ate}}}={V}_{{\rm{G}}{\mathrm{r}}}^{0}$ (diagram (i)), where ${V}_{{\rm{G}}{\mathrm{r}}}^{0}$ is the voltage required to offset any background density in the graphene. At neutrality, *ϕ*(*z*) = 0 everywhere, and the dot energy is *μ*_0_ (we note that *μ*_0_ could vary depending on the type of defect^10^). The gate voltage affects the potential map by setting *ϕ*(−*d*_Gate_) = −e*V*_Gate_, *d*_Gate_ being the thickness of the gate dielectric, and e the absolute value of the electron charge. Applying positive gate voltage *V*_Gate_ > 0 negatively charges the graphene layer. Throughout the measurement, graphene electrochemical potential eV_Gr_ is kept at ground:  −e*V*_Gr_ = e*ϕ*(0) + *μ*_Gr_ = 0, and hence the accumulation of negative charge results in *μ*_Gr_ > 0 and a downward shift in *ϕ*(0).

Changing the graphene chemical potential *μ*_Gr_ modifies the dot energy, as is seen in diagrams (ii, iii). Here, an electric field *E* = −d*ϕ*/d*z* penetrates to the gap between graphene and the source electrode at position *z* = *d*_Src_. The dot, residing at position *z* = *d*_D*ot*_ will change it chemical potential to *μ*_Dot_ = e*ϕ*(*d*_Dot_) + *μ*_0_. Current will flow through the dot only if 0 ≤ *μ*_Dot_ ≤ −e*V*_Src_ or 0 ≥ *μ*_Dot_ ≥ −e*V*_Src_. Conduction onset at zero-bias will take place when the dot is resonant with both graphite source and graphene drain (diagram (iii)). In diagram (iv) we plot the condition of current onset at finite *V*_Src_ where  −e*V*_Src_ = *μ*_Dot_. Here *V*_Src_ < 0 is applied concomitantly with *V*_Gate_ > 0 to keep graphene density constant.

The source onset condition, where the dot potential is resonant with the source, is sensitive to changes in graphene chemical potential *μ*_Gr_. This is seen by writing the dot potential as (Supplementary Note 1)

1 $${\mu }_{{\rm{D}}{\mathrm{ot}}}={\mu }_{0}+{\mu }_{{\rm{G}}{\mathrm{r}}}\left(\frac{{d}_{{\rm{D}}{\mathrm{ot}}}}{{d}_{{\rm{S}}{\mathrm{rc}}}}-1\right)-{\rm{e}}{V}_{{\rm{S}}{\mathrm{rc}}}\frac{{d}_{{\rm{D}}{\mathrm{ot}}}}{{d}_{{\rm{S}}{\mathrm{rc}}}}$$

Using the source-onset condition, we find that *V*_Src_ traces *μ*_Gr_ up to a constant along the source onset line:

2 $${V}_{{\rm{S}}{\mathrm{rc}}}=\frac{{\mu }_{{\rm{G}}{\mathrm{r}}}}{{\rm{e}}}+\frac{{\mu }_{0}}{{\rm{e}}}\frac{{d}_{{\rm{S}}{\mathrm{rc}}}}{{d}_{{\rm{D}}{\mathrm{ot}}}-{d}_{{\rm{S}}{\mathrm{rc}}}}$$

As we show in Supplementary Eq. (11), this condition can be used to extract compressibility from the slope of the source onset line. As expected for single-layer graphene, the source-onset line *V*_Src_ (*V*_Gate_) traces a square-root dependence.

In Fig. 1c we identify the transport signatures of two distinct dots (Dot 1 and Dot 2), which likely reside in different regions and conduct in parallel (upon a 2^nd ^cooldown, Dot 1 has shifted in gate voltage with respect to Dot 2). From Supplementary Eq. (11), the maximal slope (absolute value), marked in the figure, is found where the graphene layer near each dot reaches the charge-neutrality point (CNP). Dot 1 has lower *μ*_0_, and is accessible at lower density. Its charging energy, estimated from the crossing marked by a red arrow in the figure, is *U* ~ 110 meV. We note that this is a higher value than found in similar studies^34,37^. Dot 2 appears at a higher density. It exhibits an energy splitting even at *B* = 0 T (the origin of which is not presently understood).

Transport through the barrier dots is simulated by calculating a capacitive model of double-gated graphene^38^ with a fermi velocity of *v*_F_ = 1.1 × 10^6^ m s^−1^ resulting in the differential conductance map shown in Fig. 1d. In this model, capacitive charging is induced by the source and gate voltages following:

3 $$-e{n}_{{\mathrm{Gate}},{\mathrm{Src}}}={C}_{{\mathrm{Gate}},{\mathrm{Src}}}\left({V}_{{\mathrm{Gate}},{\mathrm{Src}}}-\frac{{\mu }_{{\mathrm{G}}{\mathrm{r}}}}{{\rm{e}}}\right)$$

Where *n*_Gate,Src_ are the charge densities accumulated on the gate and source electrodes respectively, *C*_Gate,Src_ are the corresponding capacitances. The total charge is fixed to an initial *n*_0_.

4 $${n}_{{\rm{G}}{\mathrm{ate}}}+{n}_{{\rm{S}}{\mathrm{rc}}}+{n}_{{\rm{G}}{\mathrm{r}}}={n}_{0}$$

and *n*_G*r*_ is related to *μ*_Gr_ by an integral on the DOS, *ρ*(*E*):

5 $${n}_{{\rm{G}}{\mathrm{r}}}=\mathop{\int}\nolimits_{0}^{{\mu }_{{\rm{G}}{\mathrm{r}}}}\rho (E)dE$$

Together, Eqs. (3)–(5) yield an integral equation for *μ*_Gr_:

6 $$\mathop{\int}\nolimits_{0}^{{\mu }_{{\rm{G}}{\mathrm{r}}}}\rho (E)dE={n}_{0}+\frac{{C}_{{\rm{G}}{\mathrm{ate}}}{V}_{{\rm{G}}{\mathrm{ate}}}+{C}_{{\rm{S}}{\mathrm{rc}}}{V}_{{\rm{S}}{\mathrm{rc}}}}{{\rm{e}}}-\frac{{\mu }_{{\rm{G}}{\mathrm{r}}}}{{{\rm{e}}}^{2}}({C}_{{\rm{G}}{\mathrm{ate}}}+{C}_{{\rm{S}}{\mathrm{rc}}})$$

By numerically solving Eq. (6) while using the known DOS of graphene $\rho =\frac{2}{\pi {v}_{{\rm{F}}}^{2}{\hslash }^{2}}| E|$, we find *μ*_G*r*_(*V*_Gate_, *V*_Src_). Extracting *μ*_Dot_ using Eq. (1), we obtain a differential conductance map for the contribution of each dot. From the simulation we extract *μ*_0_ = 40 meV for Dot 1 and *μ*_0_ = 67 meV for Dot 2. For both dots *d*_Dot_  = 1 nm. Although we have no information about the chemical identity of the defects, the capacitive model places both of them at the center of the five layers.

### Landau level spectroscopy

**Fig. 2:**
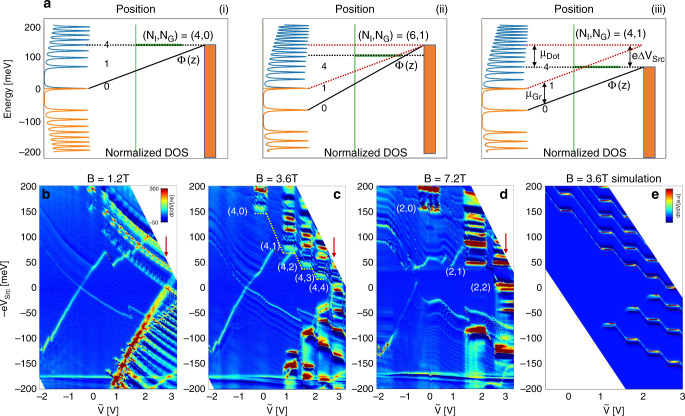
**Tunneling through a quantum dot at finite magnetic field**. **a** Energy diagrams describing dot energy (black dashed line) with respect to graphene spectrum (orange—occupied levels, blue—unoccupied levels). Panels (i)–(iii) correspond to the transition from (*N*_I_, *N*_G_) = (4, 0) to (4, 1). The transition from (i) to (iii) is decomposed into two steps: Constant bias (i, ii) where (*N*_I_, *N*_G_) changes from (4, 0) to (6, 1), and constant density (ii, iii) where the injection level is recovered ((*N*_*I*_, *N*_*G*_) = (4, 1)). The red dashed line reflects *ϕ*(*z*) and *μ*_D*ot*_ at (i). Variation in e*V*_Src_ between (i) and (iii) equals the change in *μ*_G*r*_. **b**–**d** d*I*/d*V* maps at *B* = 1.2 T, *B* = 3.6 T and *B* = 7.2 T. The dashed line in **c** marks a simulated trajectory where injection level remains constant while varying the ground state. The horizontal axis shows $\tilde{V}$, a linear combination of *V*_Src_ and *V*_Gate_ such that the density is constant along vertical lines. Red arrows mark $\tilde{V}=2.8$ V which corresponds to *n*_G*r*_ = 3.1 × 10^12^ cm^−2^, where Fig. 3a is measured. **e** Simulated d*I*/d*V* map at *B* = 3.6 T shows the same step structure as the data. The steps corresponding to illustrations (i)–(iii) in **a** are marked.

We now turn to the effect of perpendicular magnetic field *B* on the transport through the quantum dot. In Fig. 2b–d, we plot d*I*/d*V* maps while applying magnetic fields of *B* = 1.2, 3.6, and 7.2 T, respectively. The horizontal axis shows $\tilde{V}={V}_{{\rm{G}}{\mathrm{ate}}}+\frac{{C}_{{\rm{S}}{\mathrm{rc}}}}{{C}_{{\rm{G}}{\mathrm{ate}}}}{V}_{{\rm{S}}{\mathrm{rc}}}$ —a linear combination chosen such that graphene equal density lines are vertical. The same data, presented vs. *V*_Gate_, appear in Supplementary Fig. 1. At the quantum Hall regime the onset of the dot transport attains a step-like structure in the ($\tilde{V}$, −e*V*_Src_) plane, characterized by flat conductance features whose width along the $\tilde{V}$ axis increases due to increasing Landau level degeneracy. To further elucidate the structure of these features, we focus on Dot 2, where steps are sharpest.

The origin of the step structure is in the Landau level DOS, as seen in Fig. 2(a) which depicts the energy diagram at a finite magnetic field. The DOS in the quantum Hall regime consists of discrete energy levels broadened due to disorder. In this regime, the onset of electron injection into graphene takes place when *μ*_Dot_ < −e*V*_Src_ and at the same time the dot is resonant with an unpopulated Landau level: −*μ*_Gr_ + *E*_*N*_ = *μ*_Dot_. A similar condition holds for electron ejection at the opposite bias. Panel (i) depicts such a resonant condition with electrons injected into Landau level *N* = 4. At the same time, the graphene Fermi energy resides within the *N* = 0 Landau level. Since the measured spectrum depends both on the graphene ground state, and on the injection state, which are generally not the same, we designate each spectral feature by the respective pair of ground state and injection indices (*N*_I_, *N*_G_). *N*_I_ > *N*_G_ (*N*_I_ < *N*_G_) for injection (ejection) of electrons.

The *N*_G_ = 0 feature of Dot 2 is found at ($\tilde{V}=0$,  −e*V*_Src_ = 155 meV) at all magnetic fields. The dashed line in Fig. 2c is the source-onset line in this field. Along this line the dot injects carriers to the same excited state *N*_I_, while the graphene electron density increases such that *N*_G_ changes from 0 to 4. This trajectory can be traced until *V*_Src_ = 0, where the dot is resonant with the source and the drain, and electrons are injected into the ground state. At *V*_Src_ = 0 we have *N*_I_ = *N*_G_, identifying *N*_I_ = 4 for this entire trajectory.

The (4,0) feature thus corresponds to the compressible regime, where *μ*_Gr_ resides within the 0^th^ Landau level, the DOS is very large, and graphene perfectly screens the electric field applied by the gate. Once this level is filled, graphene enters the incompressible regime. Its carrier density can not change—allowing almost perfect field penetration. As a result, *ϕ*(*z* = 0) shifts sharply downwards. The next compressible regime, corresponding to the ground state *N*_G_ = 1, appears at  −e*V*_Src_ = 78 meV.

Between (4, 0) and (4, 1), the graphene ground state changes while the dot is kept resonant with the same injection level. Using Eq. (1) we show in Supplementary Notes 2 and 3 that along such a trajectory Δ*μ*_Gr_ = eΔ*V*_Src_, where Δ*μ*_Gr_ is the change in graphene chemical potential, and Δ*V*_Src_ is the change in *V*_Src_ required to keep the same Landau level resonant. Using this relation, we find that the difference between the *N*_G_ = 0, 1 plateaus is 77 meV, in agreement with the parameters used for the fit in Fig. 1d. More generally, we can simulate the entire spectrum using the same model developed above (Eq. 6), while assuming a density of states consisting of Gaussian-broadened Landau levels. From the simulation we extract the source onset line corresponding to the *N*_I_ = 4 trace. Plotted as a yellow dashed line in Fig. 2c, this trace agrees very well with experimental data.

### Degeneracy lifting

The correspondence between *V*_Src_ and *μ*_Gr_ (Eq. 2) shows that the source-onset line is sensitive to changes in *μ*_Gr_ and can hence be used as a compressibility probe (Supplementary Eq. 11). In this sense, the barrier-defect functions as a very local single-electron transistor (SET)^39–42^. In what follows, we show that the dot also serves as a spectral probe, sensitive to excited state DOS. The dot is used as a spectrometer by keeping *n*_Gr_ constant and scanning the injection energy, as explained schematically in panels (ii, iii) of Fig. 2a. Here, when *n*_Gr_ is kept constant by balancing *V*_Gate_ and *V*_Src_, the dot energy follows Δ*μ*_Dot_ =  (*d*_Dot_/*d*_Src_)Δ*V*_Src_. Thus, keeping the ground state fixed, the dot maps the spectra of different injection levels *N*_I_.

**Fig. 3:**
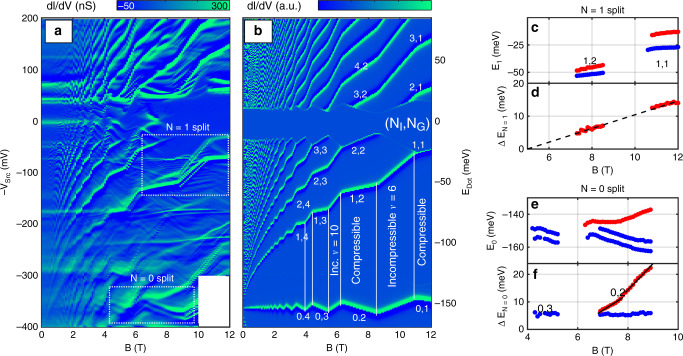
**Spectrum at fixed density**. **a** d*I*/d*V* vs. −*V*_Src_ and *B*, graphene density fixed at *n*_Gr_ = 3.1 × 10^12^ cm^−2^. Dashed regions mark *N* = 0 and *N* = 1 spectral features which show evidence of degeneracy lifting. **b** Simulation modelling the data at **a**. *μ*_Dot_ = 95 meV, *d*_Dot_ = 0.85 nm. Spectral features are marked by index pair: Injection/ejection level *N*_I_ and ground state *N*_G_. Compressible and incompressible regimes are marked at the negative bias. **c** Energies of the split peaks of the *N* = 1 feature. **d** Energy difference of the features in **c**. Dashed line corresponds to *g* = 36. **e** Energies of the split peaks of the *N* = 0 feature. **f** Energy difference of the peaks in **e**. Blue: Valley split. Red: Spin split. Overlay lines trace two different slopes, corresponding to *g* = 100 and *g* = 160, respectively.

Barrier-dot-assisted spectroscopy is demonstrated in Fig. 3a, where density is kept fixed at *n*_Gr_ = 3.1 × 10^12^ cm^−2^ near dot 2 (marked by arrows in Fig. 2b–d). *V*_Src_ is scanned from  −400 to  +200 mV, corresponding to dot energies *E*_Dot_ = −180 to 90 meV (axis on the right of b). The spectrum is dominated by Landau levels whose energies follow the well known $\sqrt{B}$ dependence, with kinks appearing as the graphene ground state shifts between compressible and incompressbile regimes. The simulation (panel b), based on the same model used above, reproduces this spectral map with excellent fidelity. The sharply peaked dot DOS causes the spectra measured this way to be extremely stable, since the DC contribution from levels below the dot energy is strongly suppressed. Compared to spectra measured using STM^9,43^, it is seen here that the dot-assisted tunneling produces clear spectra at high bias, with well-resolved Landau levels at energies well over 150 meV. As we show below, the use of this probe on high quality encapsulated graphene samples reveals energy splitting related to the SU(4) degeneracy lifting in excited states.

In Fig. 3a, two regions are marked, where the observed spectrum deviates from the calculation appearing in b. In these regions, which correspond to injection of holes to the *N*_I_ = 1 and *N*_I_ = 0 levels, the spectral features are split due to lifting of the fourfold spin-valley degeneracy. The split spectral features of the *N*_I_ = 0 state are extracted and plotted separately in panel e. The spectrum consists of two peaks, which exhibit a separation of 6 meV visible at fields as low as *B* = 4  T. This split remains fixed all the way to 9 T—suggesting a spin-independent origin. Since in *N*_G_ = 0 the valley and sub-lattice degrees of freedom are coupled, this split could be due to a local breaking of sub-lattice symmetry—e.g. due to substrate effects, which should be independent of magnetic field.

At 6 T, a third spectral feature becomes visible. This feature, marked in red in panel e, opens a gap which broadens rapidly (panel f). The gap, which evolves linearly with magnetic field, reaches a value of 17 meV at 9 T. It exhibits a very high slope: Extracting the *g* factor using Δ*E*_Z_ = *gμ*_B_*B* (where *μ*_B_ is the Bohr magneton), the observed split corresponds to *g* ≈ 100 for 7 < *B* < 8 T, and *g* ≈ 160 for 8 < *B* < 9 T (Fig. 3f). We can compare these values to STM measurements. For single-layer graphene on SiC^26^, the *N* = 0 gap stands at 20  meV at 13 T. Another study, on tri-layer graphene, finds *g* = 14.^44^ Here we find that in our device the gap is both larger, and develops faster in magnetic field. Another large splitting appears at the *N*_I_ = 1 feature. Extracting the split peak energies (Fig. 3c), we find an energy gap following a linear dependence with *g* =  36 (Fig. 3d).

The origin of such large Zeeman splits is puzzling. In atomic defects *g* ≈ 2^11^, so the split is hence likely to originate in the graphene layer. Quantum Hall states are known to exhibit strong enhancements of the *g* factors, associated with exchange coupling^45,46^. *g* may vary with sample quality, increasing in cleaner samples where a larger number of carriers may be polarized^23^. The exchange energy split, predicted in ref. ^45^ is  ≈40 meV at 10 T—not far from the values we measure. Here, both the *N*_I_ = 1 and *N*_I_ = 0 states exhibit a split feature, which extrapolates to zero at *B* = 5 T, where *N*_G_ = 3. We thus speculate that the observed splitting is related to an interplay between the state of the injected carriers, and the onset of a spontaneous polarization at the *N*_G_ = 3 state. In this scenario, the excited holes injected at the *N*_I_ = 0, 1 state undergo a strong exchange interaction with polarized spins in the ground state. This is plausible, since carriers at different Landau levels occupy the same spatial coordinates. Alternatively, the barrier dot which is 1 nm away from the graphene layer may itself experience strong exchange coupling to the graphene layer. Distinguishing between these two models requires calculations which are beyond the scope of the present work.

**Fig. 4:**
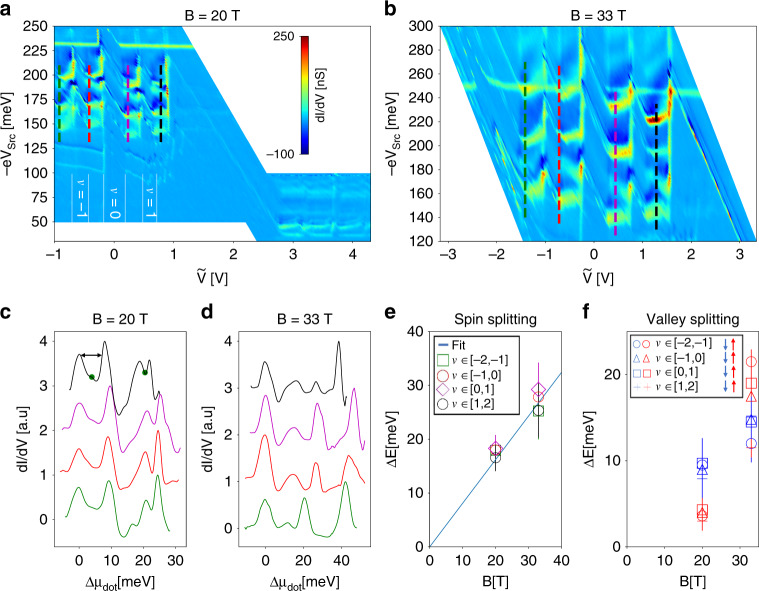
**Degeneracy Lifting at High Fields**. **a** (*N*_I_ = 1, *N*_G_ = 0) (upper left) and (*N*_I_ = 1, *N*_G_ = 1) (lower right) at *B* = 20 T. Splitting along the $\tilde{V}$ axis corresponds to degeneracy lifting of the ground state, while splitting along the  −e*V*_S*rc*_ axis corresponds to splitting of the injection state. Regions of integer filling factor are marked. **b** (*N*_I_ = 1, *N*_G_ = 0) at *B* = 33 T. **c**, **d** d*I*/d*V* vs. dot energy along the colored lines in **a**, **b**. The means of the low and high bias pairs of peaks are marked by green dots. The energy difference within each pair is marked by the double-headed arrow. **e** The energy difference between the mean of the low and high energy two peaks, for every colored line (fill factor) in panels a,b. Error bars correspond to half the peak width. The differences are plotted along a linear fit to the Zeeman energy splitting with a fit to *g* = 14 ± 1. **f** The energy difference between same-spin peaks vs. *B*. Blue markers indicates the down-spin peaks, and red markers indicate the up-spin peaks.

Turning our attention to energy splitting where the *N* = 0 level is tuned to the ground state, we notice that the feature marked by (*N*_I_ = 2, *N*_G_ = 0) in Fig. 2d already shows incipient signatures of degeneracy lifting at *B* = 7.2 T. Further increase of the magnetic field resolves the structure of the (*N*_I_, 0) feature. The data, presented in Fig. 4, were taken at the high magnetic field facility in Grenoble at temperature *T* = 1.2 K. In panel a we plot the spectra measured at *B* = 20 T. We find spectral features corresponding to *N*_G_ = 0 and *N*_G_ = 1. We focus on the *N*_G_ = 0 manifold, where the continuous feature found at lower fields has separated into an intricate pattern consisting of 16 distinct features. The origin of this structure is in the four-fold degeneracy lifting of both ground state *N*_G_ and injection state *N*_I_: As we’ve seen in Fig. 3, tuning *V*_Src_ maps the excited state spectrum at the injection level—in this case *N*_I_ = 1. At this high magnetic field, all four levels are distinguishable—as seen also at the fixed-density line-cuts, presented in panel c. Along the horizontal ($\tilde{V}$) axis, the breakup into four features is a consequence of degeneracy lifting in the *N*_G_ = 0 ground state. The spectrum clearly exhibits narrow incompressible regions, corresponding to fill factors *ν* = −1, 0, 1.

To understand which degeneracy drives the dominant splitting, we measure the energy difference between the mean of the two low energy peaks and the mean of the two high energy peaks of the spectra in Fig. 4c, d. The differences between these means (green dots in panel c), Δ*E*, are plotted in Fig. 4e. For each of the available magnetic field data sets (B = 20, 33 T), we have four data points—corresponding to the different ground states  ±*ν* ϵ [1, 2] and  ±*ν* ϵ [0, 1]. As seen in panel e, at each magnetic field all four points are bunched closely together. A single fit for Δ*E*(*B*) can be used for all fill factors. The fit follows a straight line which extrapolates to zero, implying that Zeeman splitting is the leading degeneracy lifting term, in agreement with early transport measurements^24,25^. The slope yields *g* = 14 ± 1. Splitting the *N* = 1 level should be compared to transport results with a ground state fill factor *ν* = 4. We find a greater *g* factor than those found before, where excitations at *ν* = −4 yielded *g* = 7^23^. A number of causes could explain this difference. First, in ref. ^23^, the gap is measured via temperature dependence of a macroscopic sample, with *N* = 1 being the ground state. Here, we measure the spectrum of an excited state, with *N* = 0 being the ground state. Second, our measurement is local, and is hence less prone to averaging effects of disorder.

Having identified the leading split with the Zeeman term, we associate the two lower energy states with spin up, and the two higher energy states with spin down. In Fig. 4f we plot the energy difference within each same-spin pair. It is evident that in all fill factors, the gap between spin-down states (blue markers) increases, but by a lesser amount with respect to the gap between spin up states (red markers). This result indicates that the valley splitting depends on the spin state, suggesting a coupling between the valley and spin degrees of freedom. Coupling between spin and valley degeneracy lifting has been discussed in graphene quantum dots localized by STM tips^27,47,48^, where strong confinement lifts orbital degeneracy. Our system lacks a strong confining potential. Even if the dot is charged, the charging field should be screened at the compressible limit.

Here we suggest that valley splitting could be indicative of the nature of the ground state. In the Anti-ferromagnetic (AF) or canted anti-ferromagnetic (CAF) ground states, for example, spin is coupled to the sub-lattice degree of freedom, which translates to the valley degree of freedom at *N* = 0. In Fig. 4, electrons are injected into the *N*_I_ = 1 level while *N*_G_ = 0. We speculate that for the single-particle state of the injected spin-polarized electron, the valley degree of freedom will dictate its relative overlap with the spins of the many-body ground state. While this calls for further calculation, it is likely the ferromagnetic (F) state can be ruled out when such splitting is observed.

## Discussion

Based on the compiled values of degeneracy lifted energy splitting (Table 1) we can conclude that exchange interactions appear to play a major role in the spin-split state in high quality samples. The spin-split features measured in Fig. 3 develop within a very small window in magnetic field—suggesting some pre-condition for their onset. In addition, we find that the ground state plays an important role in determining the excited state spectrum. The *N*_I_ = 1 feature develops differently when the ground state is *N*_G_ = 0 (*g* = 14) and when *N*_G_ = 1, 2 (*g* = 36). This could be possible if, indeed, the splitting is governed by exchange interactions—since the injected/ejected carrier will experience the strongest interactions with carriers in the ground state. Finally, we find that a non-trivial interplay exists between spin and valley splitting.

**Table 1:** Compilation of *g* values measured for degeneracy lifted states.

*N*_G_	*N*_I_	*g*	*B*(*T*)	
2,3	0	100–160	4–9	Fig. 3
1,2	1	36	6–12	Fig. 3
0	1	14	20–33	Fig. 4

At high magnetic fields, we also notice that the barrier dot becomes sensitive to the transition between compressible and incompressible regimes. As seen in Fig. 4a, b, upon filling each Landau level the dot energy shifts vertically up before turning down again. We suggest that the sharp evolution of the density-dependent dot spectrum is a consequence of dot sensitivity to local disorder potential, which forms electrically floating regions in the graphene layer. Since the observed feature appears close to the full Landau level limit, the floating compressible island should be hole-like, and is coupled to a local potential maximum. Interestingly, such maximum could be induced by the negative charge of the dot itself^48,49^. Confirming this, however, will require further study with additional samples.

Our results demonstrate the efficacy of barrier-dot-assisted spectroscopy as a probe for graphene in strongly interacting quantum Hall states. The wide values of energy splitting away from the ground state suggest that strong exchange interaction has to be considered between excited carriers and polarized ground states. The observation of such features indicates that the barrier dot, as a probe, retains fragile many-body states. This could be the consequence of minimal screening, planar geometry, or both. As barrier dots may also serve as local sensors for the chemical potential, they thus merge the capabilities of local SETs^39–42^ with probes which retain planar geometry^50^. Their size, positioning and non-invasiveness thus make barrier dots potentially useful probes for other interacting systems.

## Methods

### Device fabrication

The vdW tunnel junction was fabricated using the vdW transfer technique. The graphite flake was exfoliated on a SiO_2_ substrate. The hBN barrier and graphene flakes were transferred on top of the graphite flake, respectively. On top of the graphene flake, another bulk hBN flake was transferred, with the purpose of acting as a gate dielectric. Ti/Au contacts were fabricated using standard electron beam lithography techniques. Contact evaporation was executed at high vacuum and at  −5 °C.

## Supplementary information

Supplementary Information

Peer Review File

## Data Availability

The data that support the findings of this study are available from the corresponding author upon request.
