# Supplementary material for: Quantum-dot assisted spectroscopy of degeneracy-lifted Landau levels in graphene
Source: Nat Commun. 2020 Jul 8;11:3408. doi: 10.1038/s41467-020-17225-1 (PMC7343833; doi:10.1038/s41467-020-17225-1)
Supplement: Supplementary file 1 — Supplementary Information [file 41467_2020_17225_MOESM1_ESM.pdf]

REVIEWER COMMENTS

Reviewer #1 (Remarks to the Author):

Keren et al. introduce a novel and very interesting spectroscopic technique for the study of layered 2D van der Wall's devices and apply it to monolayer graphene. Using a very thin hBN dielectric they obtain a tunneling current, between the graphene and a graphite bottom gate which serves as a source, only through individual hBN defects acting as quantum dots. This allows them to probe the local density of states in the sample in a manner not wholly dissimilar to STM, but without the seemingly unavoidable tip gating of the latter technique. The results which they on the splitting of the Landau levels obtain at high magnetic fields are also of significant interest.

The explanation of their tunneling setup seemed to me quite thorough and detailed, and backed by algebra which is rather easy to understand and thoroughly worked out in the supplementary information. One minor quibble is that in Fig. 1(a) the source voltage is labeled V_SD which might lead to confusion, as in the text this is uniformly designated V_Src.

There are some things which I wish the authors could elaborate. One thing which I think would be useful to workers would be related to the frequency of occurrence of these quantum dots in the hBN. In Fig. 1 is shown data in which (as the simulation makes especially clear), the existence even of a second dot significantly complicates the observations; ideally one would have only one dot whose level participates in the tunneling. One supposes that these dots must occur in a somewhat random pattern depending on the quality of the hBN, but on average there should be a certain number of them per unit area and for this purpose it would be useful to know what was the actual area of the thin hBN barrier in this experiment; if it was in the paper I must have missed it. (Even a microscope picture of the device, perhaps added to the supplement, would help here.) If the authors would go further and try to estimate based on their experience how "lucky" one has to be in order to find a thin hBN flake with only one or two defects through which tunneling occurs, or the procedures used to obtain such a flake with the greatest likelihood, it would be useful for specialists.

Another question that comes to mind is if the authors could estimate (perhaps via a calculation) to what degree a circular potential in the graphene might be induced by the presence of the dot, and what effects this would have on the experimental data. (e.g., could any of the features in the experimental plots be explained by such degeneracy lifting? I am thinking for example of the flock of nearly horizontal peaks within the N=1 box of Fig. 3(a)). In any case I suppose the circular potential from the dot should be less overbearing than that of an STM tip, and does not compromise the utility of the new technique.

In summary, I find the technique and also the experimental data very interesting and worthy of publication, and I hope the authors can address some of the above technical points.

Reviewer #2 (Remarks to the Author):

This paper provides a defect-dot-assisted tunneling method to probe the Landau level spectroscopy of graphene and reveals energy split in the N = 0 and N = 1 excited Landau levels in the presence of a magnetic field. An energy split with Landé g-factor as high as 160 is observed in the N = 0 excited Landau level, and a split with g=36 is measured in the N = 1 excited level. Under high magnetic fields at 20 T and 33 T, four distinct split-levels also with large split gaps are observed and are attributed to the four-fold degeneracy lifting of graphene. The authors claim that the large splitting in energy levels is a consequence of enhanced exchange interactions.

The provided experimental technique is quite interesting, and the analysis is of very high quality. I

am inclined to recommend its publication to Nature communications. However, I would like to urge the authors to address the following issues before I can make my recommendation.

1. How thick the hBN barrier used in experiment is? It is that mentioned in page 7 "We find the barrier thickness to be dSrc = 2 nm, likely 3 layers of hBN"? I think it would be better to describe it more clearly in the beginning or in the Device Fabrication.

2. Are all the experimental data measured in one sample? Did the authors find similar results in other samples with different hBN barrier thickness? What's the effect of the barrier thickness on this tunneling measurement?

3. Why do dot1 and dot2 show so different results in Fig.1 and Fig.2？Whether it means that the dot-assisted tunneling depends on different dots? If so, depending on what parameters of the dot?

4. The valley splitting is nearly constant from 4T to 10T for the N=0 level in Fig. 3(f), and it shows an increase from 20T to 33T for the N=1 level in Fig. 4(f). What results in such a difference? And what's the origin of the valley splitting observed in this experiment?

5. In the first paragraph of page 15, the authors state that "It is evident that in all fill factors the gap between spin-down states (blue markers) barely changes". It seems that the gap between spin-down states presented in Fig. 4(f) also shows an increase with increasing the magnetic field.

6. Recently, several STM works reported the studies of the Landau level splitting in bilayer and trilayer graphene and shown large g factor for high-bias levels. The authors should mention these reports.

Minor points:

7. The authors define the bias voltage between the graphite and graphene flakes as VSrc, but such a bias is marked by VSD in the schematic illustration Fig. 1(a). It would be better to use an identical definition.

8. For the statement "Plotted as a yellow dashed line in panel (c)" in the second paragraph of page 10, the authors should point out clearly that which figure's panel (c) they want to indicate (It should be Fig. 2(c) I think).

**Referee Response for the Manuscript**

**Quantum-Dot Assisted Spectroscopy of Degeneracy-Lifted Landau Levels in Graphene**

By Itai Keren and Co-Authors

We thank both reviewers for taking the time to read our manuscript. Below we address their comments.

**Reviewer #1 (Remarks to the Author):**

**Keren et al. introduce a novel and very interesting spectroscopic technique for the study of layered 2D van der Wall's devices and apply it to monolayer graphene. Using a very thin hBN dielectric they obtain a tunneling current, between the graphene and a graphite bottom gate which serves as a source, only through individual hBN defects acting as quantum dots. This allows them to probe the local density of states in the sample in a manner not wholly dissimilar to STM, but without the seemingly unavoidable tip gating of the latter technique. The results which they on the splitting of the Landau levels obtain at high magnetic fields are also of significant interest.**

**The explanation of their tunneling setup seemed to me quite thorough and detailed, and backed by algebra which is rather easy to understand and thoroughly worked out in the supplementary information.**

**One minor quibble is that in Fig. 1(a) the source voltage is labeled V_SD which might lead to confusion, as in the text this is uniformly designated V_Src.**

Figure 1 was corrected according to the Reviewer’s comment.

**There are some things which I wish the authors could elaborate. One thing which I think would be useful to workers would be related to the frequency of occurrence of these quantum dots in the hBN. In Fig. 1 is shown data in which (as the simulation makes especially clear), the existence even of a second dot significantly complicates the observations; ideally one would have only one dot whose level participates in the tunneling. One supposes that these dots must occur in a somewhat random pattern depending on the quality of the hBN, but on average there should be a certain number of them per unit area and for this purpose it would be useful to know what was the actual area of the thin hBN barrier in this experiment; if it was in the paper I must have missed it. (Even a microscope picture of the device, perhaps added to the supplement, would help here.) If the authors would go further and try to estimate based on their experience how "lucky" one has to be in order to find a thin hBN flake with only one or two defects through which tunneling occurs, or the procedures used to obtain such a flake with the greatest likelihood, it would be useful for specialists.**

We agree with the Reviewer that extending the utility of this technique requires increasing the probability of finding a single dot within the area capped by a single

gate. As seen in the optical image we have added (Figure S2), the surface area of the gated tunnel junction is 45 $\mu m^2$.
Our device also has an ungated region of area 30 $\mu m^2$. Based on the presence of a resonant feature appearing in Figure 1,2 at -180 meV, we believe there is a single dot in this area. We thus have, on average, one dot per 25 $\mu m^2$.
Our data originates in a single device. It is nevertheless possible to extend the statistics by tracking publications by other groups (Table R1). Greenaway et al. (Ref 10), estimate hBN dot density to be "lower than $10^{-12}$ $m^{-2}$". They report two dots on a 50 $\mu m^2$ area on one device, and a denser population on another.
Chandni et al. (2015, Ref 33) report defect-assisted tunneling in Au-hBN-Au tunnel junctions, which should originate from the same type of dots. They do exhibit dot physics in junctions a few $\mu m^2$ in area, but do not report on the number of dots. In a later paper, the same group (Ref 52) reports a single gate-tunable dot in a Au-hBN-Graphene device, though they do not report the junction area.
Interestingly, dot-assisted tunneling is also observed where direct tunneling is fully suppressed - e.g. hBN 6 layers thick. We thus conclude that the recipe for reproducing devices with transport-accessible defect dots entails the use of hBN barriers 4-6 layers thick, with gate area of approximately 20 $\mu m^2$.
*We have added this information in Supplementary Section 5.*
Finally, we note that there is a much higher probability of finding dots in transition metal dichalcogenide (TMD) barriers [Ref 11]. Here, dot density is about 2 orders of magnitude higher.

| Paper | Greenaway (Ref 10) | Chandni 2015 (Ref 33) | Chandni 2016 (Ref 52) | Our Study |
|---|---|---|---|---|
| Number of Dots | 2,50 | ? | ? | 2,1 |
| Device Area ($\mu m^2$) | 50, 25 | 0.7-8 | | 45,30 |
| Barrier Thickness | 1.5,1 | 3-6 layers | | 5 Layers |
| Energy distribution in V | ±1, ±0.25 | | | |

*Table R1: Defect dots observed in our study and prior works*

**Another question that comes to mind is if the authors could estimate (perhaps via a calculation) to what degree a circular potential in the graphene might be induced by the presence of the dot, and what effects this would have on the experimental data. (e.g., could any of the features in the experimental plots be explained by such degeneracy lifting? I am thinking for example of the flock of nearly horizontal peaks within the N=1 box of Fig. 3(a)). In any case I suppose the circular potential from the dot should be less overbearing than that of an STM tip, and does not compromise the utility of the new technique.**

The reviewer is making a very important point. Indeed, the bare local potential would vary between the charged and discharged states of the defects. In our experiment the dot population changes by 1 unit of charge - although we do not know if the initial state of the defect is neutral or positively charged.
While the neutral dot state does not affect the potential landscape, upon charging we do expect to find variation in the graphene chemical potential. To evaluate how large is the induced density in nearby graphene we rely on the experimental evidence provided by the STM studies by the Crommie group (Ref. 50) and the Andrei group

(Ref 49). In Ref 50, defects residing at close proximity to the graphene layer within the hBN substrate shift the position of the Dirac point by approximately 20-30 meV at B = 0. The effect of such potential shift on our results presented in Figure 1 would be like any other shift due to disorder potential.
At finite B, however, the effect of the dot potential on the graphene is more complex. Depending on the graphene compressibility, the charged defect would either be fully screened (at the compressible regime) or unscreened (at the incompressible regime). A sense of this effect can be seen in Luican et al., (Ref 49), where a single charge located immediately underneath the graphene layer may shift the graphene potential, as measured by the energy of the N = 0 LL, by as much as 100 meV. As graphene chemical potential is tuned into the LL, i.e. in the compressible regime, the defect potential is screened, and the change in graphene potential is minimal.
To evaluate this effect on our system, we have to recall that tunneling into the graphene layer takes place from a charged dot. Since we do not know whether the defect is a-priori at the Q = +1 or Q = 0 state, we are interested in an observable criterion which would indicate whether indeed the dot is negatively charged.
When charged, the dot should induce a local *maximum* in the potential landscape - but only within the incompressible regime. Interestingly, our data *does* show an indication for the presence of such local maximum. This is manifest in the sharp vertical features appearing at the full Landau level side in Figure 4(a,b), which we associate with compressible hole-pockets pinched off by the local potential landscape. We have already noted that these features are related to floating compressible regions in the discussion at the bottom of Page 15.
To acknowledge the possibility that such features could actually be associated with dot-induced potential, following the Reviewer's comment, we have added the following text on Page 16:
"*Since the observed feature appears close to the full Landau level limit, the floating compressible island should be hole-like, and is coupled to a local potential maximum. Interestingly, such maximum could be induced by the negative charge of the dot itself.*"

Finally, regarding the horizontal features seen in the N = 1 box in Figure 3: We believe that these are not related to the dot potential. They may be a feature related to Dot 1, or have a different, unknown origin.

**In summary, I find the technique and also the experimental data very interesting and worthy of publication, and I hope the authors can address some of the above technical points.**

We are pleased the Reviewer finds interest in our technique, and hope our responses have addressed his / her concerns.

**Reviewer #2 (Remarks to the Author):**

**This paper provides a defect-dot-assisted tunneling method to probe the Landau level spectroscopy of graphene and reveals energy split in the N = 0 and N = 1 excited Landau levels in the presence of a magnetic field. An energy split**

**with Landé g-factor as high as 160 is observed in the N = 0 excited Landau level, and a split with g=36 is measured in the N = 1 excited level. Under high magnetic fields at 20 T and 33 T, four distinct split-levels also with large split gaps are observed and are attributed to the four-fold degeneracy lifting of graphene. The authors claim that the large splitting in energy levels is a consequence of enhanced exchange interactions.**

**The provided experimental technique is quite interesting, and the analysis is of very high quality. I am inclined to recommend its publication to Nature communications. However, I would like to urge the authors to address the following issues before I can make my recommendation.**

**1. How thick the hBN barrier used in experiment is? It is that mentioned in page 7 "We find the barrier thickness to be dSrc = 2 nm, likely 3 layers of hBN"? I think it would be better to describe it more clearly in the beginning or in the Device Fabrication.**

Following the Reviewers comment, we have re-checked our evaluation of the hBN barrier thickness. Our estimate for the layer thickness is based on two observations: First, the direct tunneling conductance (not dot assisted) is ~ 300 MΩ. For a junction of area 75 $\mu m^2$ (gated + non-gated areas), this translates to 25 GΩ / $\mu m^2$. Following Britnell et al, (Nano Letters 12, 3, 1702 (2012)), we extrapolate the barrier thickness, correcting it to 5 layers.
This value agrees with the second observable, extracted from the capacitive model, which indicates the barrier thickness is 2 nm. Since the displacement between each two layers of hBN is 0.34 nm, there are 6 interlayer gaps for 5 layers. Hence a 5 layer barrier agrees with $d_{Src}$ = 2 nm in total.
We have added the following text "*The bottom (source) electrode is a graphite flake onto which a tunnel barrier hBN flake of thickness $d_{Src}$ = 2 nm (5 layers) is deposited*" in page 4. We have deleted the corresponding text from page 7.

**2. Are all the experimental data measured in one sample? Did the authors find similar results in other samples with different hBN barrier thickness? What's the effect of the barrier thickness on this tunneling measurement?**

The referee is making a very important point. Indeed, we have measured a single sample. Samples with thinner barriers, e.g. 2-3 layer hBN, (not appearing in this work), exhibit tunneling conductance of order 100 $k\Omega^{-1}$. Such samples can not be used for dot-assisted spectroscopy, as dot-assisted conductance signal is overwhelmed by the parallel term of the large-area direct graphene-graphite tunneling. One may also consider using thicker hBN, although it is not clear if such samples would provide sufficient source-dot-drain coupling. It is thus clear that such device require the use of 4-6 layer hBN, but are unlikely to work neither with thinner, nor with thicker barriers.

**3. Why do dot1 and dot2 show so different results in Fig.1 and Fig.2？Whether**

**it means that the dot-assisted tunneling depends on different dots? If so, depending on what parameters of the dot?**

Indeed, Dot 1 and Dot 2 seem to respond differently to the presence of magnetic field. We believe that the key difference between them is due to the quality of the graphene at close proximity to each dot. Near Dot 2 the graphene layer is of superior quality, and hence allows the resolution of Landau level at lower fields.

**4. The valley splitting is nearly constant from 4T to 10T for the N=0 level in Fig. 3(f), and it shows an increase from 20T to 33T for the N=1 level in Fig. 4(f). What results in such a difference? And what's the origin of the valley splitting observed in this experiment?**

The valley splitting observed in Fig. 3(f) is related to level $N_I$ = 0, while the ground states are $N_G$ = 2 and $N_G$ = 3 (see Fig. 3(b)). This means that the tunneling hole probes the 0$^{th}$ LL, but not as a ground state. In this case, the splitting may originate in breaking in the sub-lattice symmetry. This mechanism will provide a spin-independent shift between the two valleys. We have added the following line, at the end of paragraph 1 on Page 12:

"*Since in N=0 the valley and sub-lattice degrees of freedom are coupled, this split could be due to a local breaking of sub-lattice symmetry - e.g. due to substrate effects, which should be independent of magnetic field.*"

In Fig. 4(f), valley splitting is related to $N_I$ = 1, with the ground state being $N_G$= 0. Since N = 0 is now the ground state, this raises an interesting possibility for the symmetry breaking mechanism. For $N_G$= 0, the valley splitting may be coupled to the sublattice degree of freedom if the ground state assumes the anti-ferromagnetic (AF) or canted anti-ferromagnetic (CAF) order. In these cases, the spin degree of freedom is coupled to the valley degree of freedom. Since in CAF and AF, spin down and spin up are arranged differently on the two sublattices, the interaction - and injection energy - will depend on the valley quantum number of the injected electron.

We have added the following text on page 15:

"Here we suggest that valley splitting could be indicative of the nature of the ground state. In the Anti-ferromagnetic (AF) or canted anti-ferromagnetic (CAF) ground states, for example, spin is coupled to the sub-lattice degree of freedom, which translates to the valley degree of freedom at N=0. In Fig.4, electrons are injected into the $N_I$ = 1 level while $N_G$ = 0. We speculate that for the single-particle state of the injected spin-polarized electron, the valley degree of freedom will dictate its relative overlap with the spins of the many-body ground state. While this calls for further calculation, it is likely the ferromagnetic (F) state can be ruled out when such splitting is observed."

We thank the referee for raising this important point.

**5. In the first paragraph of page 15, the authors state that "It is evident that in all fill factors the gap between spin-down states (blue markers) barely changes". It**

**seems that the gap between spin-down states presented in Fig. 4(f) also shows an increase with increasing the magnetic field.**

We agree that the gap indeed changes – although at a smaller extent than the "down" spin undergoes. We have corrected the text on page 15, first paragraph, accordingly: "*It is evident that in all fill factors, the gap between spin-down states (blue markers) increases, but by a lesser amount with respect to the gap between spin up states (red markers)*"

**6. Recently, several STM works reported the studies of the Landau level splitting in bilayer and trilayer graphene and shown large g factor for high-bias levels. The authors should mention these reports.**

We are aware of the work by the group of L. He (PRB 98 045413, 2018), showing g factors of 11-14 on trilayer graphene. A citation to this work was added on Page 12. Otherwise, high bias results were reported in refs 46-48, but these are affected by local STM tip potential distortion, and hence, we believe are not relevant for comparison.

**Minor points:**

**7. The authors define the bias voltage between the graphite and graphene flakes as VSrc, but such a bias is marked by VSD in the schematic illustration Fig. 1(a). It would be better to use an identical definition.**

This has been corrected

**8. For the statement "Plotted as a yellow dashed line in panel (c)" in the second paragraph of page 10, the authors should point out clearly that which figure's panel (c) they want to indicate (It should be Fig. 2(c) I think).**

This has been corrected

REVIEWERS' COMMENTS:

Reviewer #1 (Remarks to the Author):

The authors have nicely addressed the points I mentioned in my review. The new Fig. S2 has a typo in the scale bar: it says 10 um^2 where I suppose it was meant to say 10 um. This should be fixed prior to publication.

Reviewer #2 (Remarks to the Author):

The authors have answered my and the other referee's comments and questions well, in my opinion. I recommend the publication of this manuscript in Nature Communications.
