# Supplementary material for: Quantum-dot assisted spectroscopy of degeneracy-lifted Landau levels in graphene
Source: Nat Commun. 2020 Jul 8;11:3408. doi: 10.1038/s41467-020-17225-1 (PMC7343833; doi:10.1038/s41467-020-17225-1)
Supplement: Supplementary file 2 — Peer Review File [file 41467_2020_17225_MOESM2_ESM.pdf]

Supplementary Information for "Quantum-Dot Assisted Spectroscopy of Degeneracy-Lifted Landau Levels in Graphene"

I. Keren,[1] T. Dvir,[1] A. Zalic,[1] A. Iluz,[1] D. LeBoeuf,[2]
K. Watanabe,[3] T. Taniguchi,[3] and H. Steinberg[1]

[1]*Racah Institute of Physics, The Hebrew University, Jerusalem 91904, Israel*

[2]*LNCMI, Centre National de la Recherche Scientifique,*
EMFL, Université Grenoble Alpes, INSA Toulouse,
Université Toulouse Paul Sabatier, Grenoble, France

[3]*National Institute for Material Science,*
1-1 Namiki, Tsukaba 305-0044, Japan

Supplementary Note 1. Dot Potential

We calculate the dot potential assuming a grounded graphene layer, $V_{\mathrm{Gr}} = 0$. As seen in Figure 1(b) in the main text, since $-\mathrm{e}V_{\mathrm{Gr}} = \mu_{\mathrm{Gr}} + \mathrm{e}\phi(0)$, we have $\phi(0) = -\mu_{\mathrm{Gr}}/\mathrm{e}$. The electric field between the graphene and the source is:

$$E = (\mu_{\mathrm{Gr}}/\mathrm{e} - V_{\mathrm{Src}})/d_{\mathrm{Src}} \tag{1}$$

The electric potential at the dot location d_{Dot} obeys:

$$\mathrm{e}\phi(d_{\mathrm{Dot}}) = \mathrm{e}\phi(0) + \mathrm{e}Ed_{\mathrm{Dot}} = -\mu_{\mathrm{Gr}} + (\mu_{\mathrm{Gr}} - \mathrm{e}V_{\mathrm{Src}})(d_{\mathrm{Dot}}/d_{\mathrm{Src}}) \tag{2}$$

The dot energy μ_{Dot} is shifted by μ_0 from the potential at $z = d_{\mathrm{Dot}}$:

$$\mu_{\mathrm{Dot}} = \mu_0 + \mu_{\mathrm{Gr}}\left(\frac{d_{\mathrm{Dot}}}{d_{\mathrm{Src}}} - 1\right) - \mathrm{e}V_{\mathrm{Src}}\frac{d_{\mathrm{Dot}}}{d_{\mathrm{Src}}} \tag{3}$$

Supplementary Note 2. Extracting DOS from Slope

From the resonant features in Fig. 1(c), we can extract the density of states, and quantum capacitance, using two equations. The first is the integral equation (Equation (6) in the main text) for the graphene chemical potential μ_{Gr}:

$$\int_0^{\mu_{\mathrm{Gr}}} \rho(E)dE = n_0 + \frac{C_{\mathrm{Gate}}V_{\mathrm{Gate}} + C_{\mathrm{Src}}V_{\mathrm{Src}}}{\mathrm{e}} - \frac{\mu_{\mathrm{Gr}}}{\mathrm{e}^2}(C_{\mathrm{Gate}} + C_{\mathrm{Src}}) \tag{4}$$

which relates V_{Gate}, V_{Src} and μ_{Gr}. The solution to this equation defines the charge density $n_{\mathrm{Gr}} = \int_0^{\mu_{\mathrm{Gr}}} \rho(E)dE$ for any gate and bias voltage.

Along source onset line, where the dot is resonant with the source electrode, we have $\mu_{\mathrm{Dot}} = -\mathrm{e}V_{\mathrm{Src}}$. Plugging it into the source-dot resonance condition (Supplementary Equation 3) we have:

$$-\mathrm{e}V_{\mathrm{Src}} = \mu_0 + \mu_{\mathrm{Gr}}\left(\frac{d_{\mathrm{Dot}}}{d_{\mathrm{Src}}} - 1\right) - \mathrm{e}V_{\mathrm{Src}}\frac{d_{\mathrm{Dot}}}{d_{\mathrm{Src}}} \tag{5}$$

This yields the equation

$$V_{\mathrm{Src}} = \frac{\mu_{\mathrm{Gr}}}{\mathrm{e}} + \frac{\mu_0}{\mathrm{e}}\frac{d_{\mathrm{Src}}}{d_{\mathrm{Dot}} - d_{\mathrm{Src}}} \tag{6}$$

Where the last equation means that to stay on resonance, V_{Src} will trace the graphene chemical potential up to a constant. We use two equations: The integral equation, Supplementary

Equation 4, and Supplementary Equation 6. These two depend on V_{Src}, V_{Gate} and μ_{Gr}. We are interested in an expression for a slope in the (V_{Gate},V_{Src}) plane: $\mathrm{d}V_{\mathrm{Src}}/\mathrm{d}V_{\mathrm{Gate}}$. To reach this, we plug Supplementary Equation 6 into Supplementary Equation 4:

$$\int_0^{\mu_{\mathrm{Gr}}} \rho(E)dE = n_0 - \frac{\mu_{\mathrm{Gr}}}{\mathrm{e}^2}C_{\mathrm{Gate}} + \frac{1}{\mathrm{e}}C_{\mathrm{Gate}}V_{\mathrm{Gate}} + \frac{1}{\mathrm{e}}C_{\mathrm{Src}}\left(\frac{\mu_0}{\mathrm{e}}\frac{d_{\mathrm{Src}}}{d_{\mathrm{Dot}} - d_{\mathrm{Src}}}\right) \tag{7}$$

From which we isolate V_{Gate}

$$V_{\mathrm{Gate}} = -\frac{\mathrm{e}}{C_{\mathrm{Gate}}}n_0 + \frac{\mu_{\mathrm{Gr}}}{\mathrm{e}} + \frac{\mathrm{e}}{C_{\mathrm{Gate}}}\int_0^{\mu_{\mathrm{Gr}}} \rho(E)dE - \frac{C_{\mathrm{Src}}}{C_{\mathrm{Gate}}}\left(\frac{\mu_0}{\mathrm{e}}\frac{d_{\mathrm{Src}}}{d_{\mathrm{Dot}} - d_{\mathrm{Src}}}\right) \tag{8}$$

Yielding

$$\frac{\mathrm{d}V_{\mathrm{Gate}}}{\mathrm{d}\mu_{\mathrm{Gr}}} = \frac{1}{\mathrm{e}} + \frac{\mathrm{e}}{C_{\mathrm{Gate}}}\rho(\mu_{\mathrm{Gr}}) \tag{9}$$

From Supplementary Equation 6:

$$\frac{\mathrm{d}V_{\mathrm{Src}}}{\mathrm{d}\mu_{\mathrm{Gr}}} = \frac{1}{\mathrm{e}} \tag{10}$$

Thus

$$\frac{\mathrm{d}V_{\mathrm{Src}}}{\mathrm{d}V_{\mathrm{Gate}}} = \frac{1}{1 + \frac{\mathrm{e}^2}{C_{\mathrm{Gate}}}\rho(\mu_{\mathrm{Gr}})} \tag{11}$$

Supplementary Note 3. Resonant Trajectory

In the specific case where the quantum dot traces the spectrum of a Landau level, as in Figure 2, we can reach a special case of Equation (2) (Supplementary Equation 6), showing that $\Delta\mu_{\mathrm{Gr}} = \mathrm{e}\Delta V_{\mathrm{Src}}$ upon a transition between two Landau levels. We calculate the shift in V_{Src} between the two compressible plateaus corresponding to the difference between the N and $N+1$ Landau levels. This is elucidated in the three schematic energy diagrams presented in Figure 2(a) in the main text, where the trajectory from (4,0) to (4,1) is broken into two stages. First, is a transition from (i) to (ii) along a fixed V_{Src} line, graphene shifts from ground state N_{G}, with chemical potential μ_{Gr}^{N} to ground state $N_{\mathrm{G}}+1$ with chemical potential μ_{Gr}^{N+1}. From Equation (1) (Supplementary Equation 3) we find the dot energy shift is:

$$\Delta\mu_{\mathrm{Dot}}^{(\mathrm{i})-(\mathrm{ii})} = \Delta\mu_{\mathrm{Gr}}(d_{\mathrm{Dot}}/d_{\mathrm{Src}} - 1) \tag{12}$$

Here $\Delta\mu_{\mathrm{Gr}} = \mu_{\mathrm{Gr}}^{N+1} - \mu_{\mathrm{Gr}}^{N}$ is the difference in the graphene chemical potential upon increase of a single Landau level. In the second leg of the trajectory, from (ii) to (iii), the dot is

brought back to resonance with the same N_{I} through a shift in V_{Src} while keeping n_{Gr} (and μ_{Gr}) fixed. Here Equation (1) yields

$$\Delta\mu_{\mathrm{Dot}}^{(ii)-(iii)} = \mathrm{e}\Delta V_{\mathrm{Src}}(-d_{\mathrm{Dot}}/d_{\mathrm{Src}}) \tag{13}$$

The total shift in μ_{Dot} from (i) to (iii) is

$$\Delta\mu_{\mathrm{Dot}}^{(\mathrm{i})-(\mathrm{iii})} = \Delta\mu_{\mathrm{Gr}}(d_{\mathrm{Dot}}/d_{\mathrm{Src}} - 1) + \mathrm{e}\Delta V_{\mathrm{Src}}(-d_{\mathrm{Dot}}/d_{\mathrm{Src}}) \tag{14}$$

Demanding that the dot remains resonant with the same Landau level requires:

$$\Delta\mu_{\mathrm{Dot}}^{(\mathrm{i})-(\mathrm{iii})} = \Delta\mu_{\mathrm{Gr}}$$

we have

$$\Delta\mu_{\mathrm{Gr}} = \Delta\mu_{\mathrm{Gr}}(d_{\mathrm{Dot}}/d_{\mathrm{Src}} - 1) + \mathrm{e}\Delta \mathrm{V}_{\mathrm{Src}}(-d_{\mathrm{Dot}}/d_{\mathrm{Src}})$$

which yields

$$\Delta\mu_{\mathrm{Gr}} = \mathrm{e}\Delta \mathrm{V}_{\mathrm{Src}} \tag{15}$$

This relation is general for trajectories where the dot is kept in resonance with a specific spectral feature. In our case it means that tuning the system from from (i) to (iii), while changing the graphene ground state from N_{G} to $N_{\mathrm{G}} + 1$ results in $\mathrm{e}\Delta \mathrm{V}_{\mathrm{Src}} = \mu_{\mathrm{Gr}}^{N+1} - \mu_{\mathrm{Gr}}^{N}$. This is also demonstrated in panel (iii) of Figure 2 in the main text, where we find that $\phi(z)$ corresponding to state (i), plotted in red, is parallel to the new $\phi(z)$ corresponding to state (iii), plotted in black.

Supplementary Note 4. Raw Data

In the main text, the horizontal axis used in Fig. 2 and Fig. 4 shows $\tilde{V} = V_{\mathrm{Gate}} + \frac{C_{\mathrm{Src}}}{C_{\mathrm{Gate}}} V_{\mathrm{Src}}$, defined such that the graphene equal density lines are vertical - leading to easier identification of the energy difference between Landau levels. In Supplementary Figure 1 we show the raw data, where the horizontal axis is V_{Gate}. Panels (a-c) show the same measurements presented in Fig. 2 while panel (d) shows the region marked by a red square in (b), zoomed in. This representation of the data enables appreciation of the sharpness of the transition between adjacent ground states.

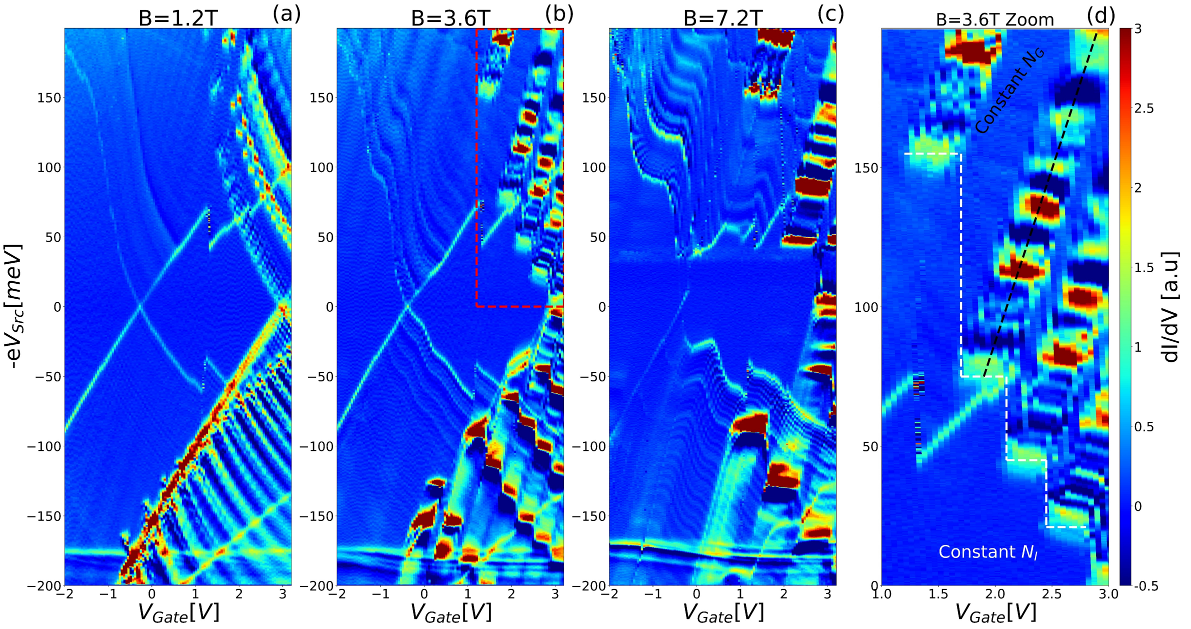

Supplementary Figure 1. Raw Data Finite Field Measurements (**a-c**) dI/dV maps at $B = 1.2$ T, $B = 3.6$ T and $B = 7.2$ T. The dashed square in (b) marks the zoomed in region for panel (d). (**d**) Zoom in on panel (b). Constant injection and constant ground state trajectories are marked (white and black respectively)

From Supplementary Figure 1(d) it is evident that the transition, along a constant injection trajectory, between adjacent ground states is vertical. This embodies the fact that once a Landau level is full, further gating the graphene with little additional charge shifts its spectrum by the energy difference between levels. Along a constant injection trajectory, V_{Src} compensates for this shift resulting in the vertical staircase structure.

Supplementary Note 5. Device Details

The data in this work originates from a single device consisting of two dots within a region of approximately 45 μm^2 pictured in Supplementary Figure 2.

In Supplementary Figure 2 one can see an overlap between graphene (white), barrier hBN (yellow) and graphite (blue) away from the gated region (red). This leads to the possibilty of un-gated dots and might explain the constant feature near -180 meV in Supplementary

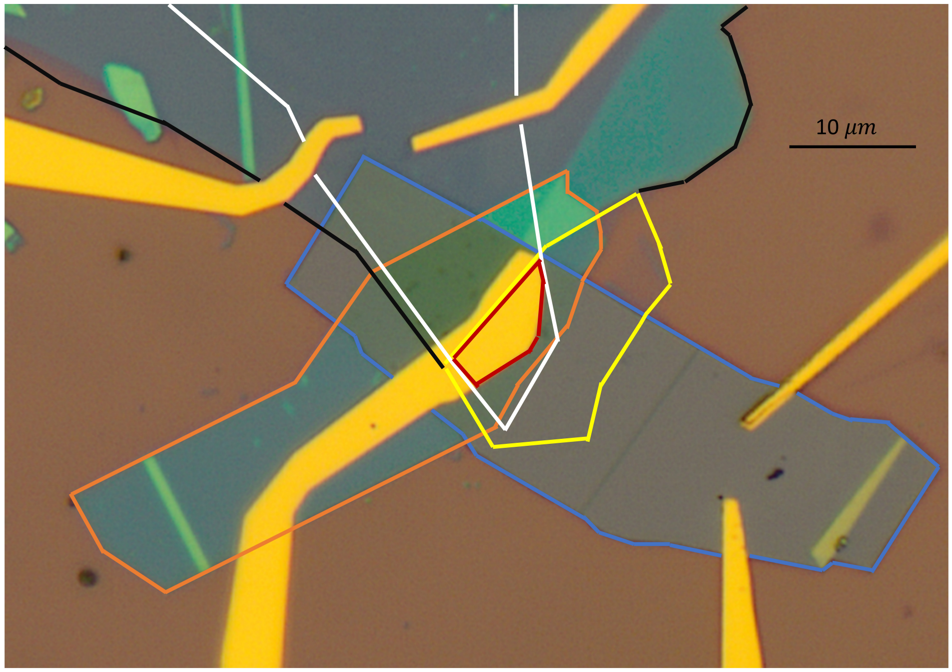

Supplementary Figure 2. Optical microscope image of the device. The different layers are outlined from bottom to top - graphite (blue), barrier hBN (yellow), graphene (white) and a top dielectric hBN (orange). The barrier hBN flake is attached to a thicker region outlined in black. The gate electrode is patterned on top of the dielectric hBN. Gated junction area is approximately 45 μm^2. The gated region is outlined in red.

Figure 1 as an additional un-gated dot. The area of the un-gated region is approximately 30 μm^2. All together, our junction has 3 dots over the region of 75 μm^2 which leads to the estimate of one dot per 25 μm^2. We note that this represents a smaller dot density than reported in Ref.[1]. Based on these dimensions, and on dimensions of devices measured by others[1–3], to obtain transport-active dots, one requires a gated junction area should be 20-30 μm^2 and barrier hBN thickness 3-6 layers.

SUPPLEMENTARY REFERENCES

[1] Greenaway, M. T. *et al.* Tunnel spectroscopy of localised electronic states in hexagonal boron nitride. *Communications Physics* **1**, 94 (2018).

[2] Chandni, U., Watanabe, K., Taniguchi, T. & Eisenstein, J. P. Evidence for Defect-Mediated

Tunneling in Hexagonal Boron Nitride-Based Junctions. *Nano Letters* **15**, 7329–7333 (2015).

[3] Chandni, U., Watanabe, K., Taniguchi, T. & Eisenstein, J. P. Signatures of Phonon and Defect-Assisted Tunneling in Planar Metal-Hexagonal Boron Nitride-Graphene Junctions. *Nano Letters* **16**, 7982–7987 (2016).
